# Targeted salivary biomarkers for discrimination of periodontal health and disease(s)

**DOI:** 10.3389/fcimb.2015.00062

**Published:** 2015-08-19

**Authors:** Jeffrey L. Ebersole, Radhakrishnan Nagarajan, David Akers, Craig S. Miller

**Affiliations:** ^1^Center for Oral Health Research, College of Dentistry, University of KentuckyLexington, KY, USA; ^2^Division of Biomedical Informatics, College of Public Health, University of KentuckyLexington, KY, USA; ^3^Department of Statistics, College of Arts and Sciences, University of KentuckyLexington, KY, USA; ^4^Division of Oral Diagnosis, Oral Medicine and Oral Radiology, College of Dentistry, University of KentuckyLexington, KY, USA

**Keywords:** periodontitis, saliva, MMP-8, cytokines, personalized medicine

## Abstract

Generally, clinical parameters are used in dental practice for periodontal disease, yet several drawbacks exist with the clinical standards for addressing the needs of the public at large in determining the current status/progression of the disease, and requiring a significant amount of damage before these parameters can document disease. Therefore, a quick, easy and reliable method of assessing and monitoring periodontal disease should provide important diagnostic information that improves and speeds treatment decisions and moves the field closer to individualized point-of-care diagnostics.

**Objective:** This report provides results for a saliva-based diagnostic approach for periodontal health and disease based upon the abundance of salivary analytes coincident with disease, and the significant progress already made in the identification of discriminatory salivary biomarkers of periodontitis.

**Methods:** We evaluated biomarkers representing various phases of periodontitis initiation and progression (IL-1ß, IL-6, MMP-8, MIP-1α) in whole saliva from 209 subjects categorized with periodontal health, gingivitis, and periodontitis.

**Results:** Evaluation of the salivary analytes demonstrated utility for individual biomarkers to differentiate periodontitis from health. Inclusion of gingivitis patients into the analyses provided a more robust basis to estimate the value of each of these analytes. Various clinical and statistical approaches showed that pairs or panels of the analytes were able to increase the sensitivity and specificity for the identification of disease.

**Conclusions:** Salivary concentrations of IL-1ß, IL-6, MMP-8, MIP-1α alone and in combination are able to distinguish health from gingivitis and periodontitis. The data clearly demonstrated a heterogeneity in response profiles of these analytes that supports the need for refinement of the standard clinical classifications if we are to move toward precision/personalized dentistry for the twenty-first century.

## Introduction

Historically, the health of the periodontium has been defined by clinical parameters that describe a lack of gingival inflammation bleeding on probing (BOP), changes in the epithelial barrier relationship to the cementoenamel junction clinical attachment level (CAL), and loss of underlying alveolar bone architecture probing pocket depth (PPD) (Armitage and Robertson, 2009; Armitage and Cullinan, 2010). However, coincident with these macro-clinical measures, histological studies have clearly identified some local tissue inflammatory response to the juxtaposed supra- and subgingival microbial ecology, even in “clinically healthy” sites (Page and Schroeder, 1976; Armitage et al., 1977; Brecx et al., 1986). This type of mucosal “physiologic inflammation” has also been described in gastrointestinal mucosal tissues and is considered important in the normal host-bacterial interactions to maintain tissue homeostasis (Silva et al., 2007; Rabinowitz and Mayer, 2012). These histological findings of the periodontium are coincident with data describing detectable levels of a select group of host response molecules in healthy tissues, that are generally considered to increase significantly in magnitude and expand in an array of responses with gingivitis (Offenbacher et al., 2009, 2010; Jönsson et al., 2011; Leishman et al., 2013) and periodontitis (Kim et al., 2006; Beikler et al., 2008; Kebschull et al., 2013, 2014). However, these findings have not been effectively extrapolated into creating a paradigm that integrates biological and clinical measures of health of the gingival tissues as an important, and potentially crucial, biomedical informatics approaches for assessing disease presence, prognosis, and progression.

Gingivitis is an often-overlooked disease, despite being the “Gateway” to periodontitis for a significant portion of the population (Page, 1986; Albandar et al., 1998; Schätzle et al., 2003, 2004, 2009; Lang et al., 2009). This issue has persisted for years, in part because clinical parameters along with pre-defined thresholds of inflammation and measurable tissue destruction have been used as the “gold standard” for discerning health/gingivitis from periodontitis. While very helpful, they neither provide insights into patient-specific variations within these inexact disease groups, nor do they help predict non-responders and those who “at risk” for disease progression. Thus, novel methods for identifying those “at risk persons” are needed.

Differential host responses are thought to contribute to various susceptibilities that play an important role in determining progression of the inflammatory lesion (Kornman et al., 1997; Trombelli, 2004; Van Dyke and Sheilesh, 2005; Grigoriadou et al., 2010; Ebersole et al., 2013). At the cellular level, exposure to bacterial products and lipopolysaccharide (LPS) elicit activation of monocytes/macrophages that promote secretion of cytokines and inflammatory mediators such as IL-1β, IL-6, and TNFα that results in the release of matrix metalloproteinases (MMPs) that undermine the integrity of the gingival tissues (Yucel-Lindberg and Båge, 2013). Many of these inflammatory molecules have been detected in oral fluids (Sorsa et al., 1999; Miller et al., 2006), which has allowed saliva to emerge as an important and easily accessible biological fluid that can provide important diagnostic information regarding oral health and disease (Henskens et al., 1993; Fine et al., 2009; Giannobile et al., 2009; Miller et al., 2010; Kinney et al., 2011; Shaila et al., 2013; Prakasam and Srinivasan, 2014) Consistent with this, recent data from our lab and others indicate that salivary concentrations of IL-6, IL-8, albumin, calprotectin, PGE_2_, MMP-8, and MIP-1α are elevated in patients who have gingivitis (Lee et al., 2012; Syndergaard et al., 2014).

Use of salivary biomarkers in conjunction with the expanded panel of potential biomarkers from recent investigations using proteomic and transcriptomic analyses could help dentistry move toward the era of personalized medicine. However, advances will require studies that analyze biospecimens and compare biomarkers from patients exhibiting the full spectrum of disease (health, gingivitis, and periodontitis), as studies regarding this spectrum of disease have been lacking. This report addresses this gap and describes an approach that reflects Phase I standards that are articulated regarding the discovery, validation, and utility assessment of biomarkers for disease detection (Pepe et al., 2001).

Periodontal disease is a chronic inflammatory and destructive condition that affects an estimated 80% of U.S. adults that can have significant systemic consequences. Customary clinical parameters are used in dental practice because of their ease of use, relative non-invasiveness and reliability. Yet several drawbacks exist with the current standards for addressing the needs of the public at large. First, a highly trained clinician and assistant are needed to record the findings. Second, collection of this diagnostic information includes the use of expensive radiographic equipment that makes the procedure time and labor intensive, as well as imposing significant financial costs to the consumer. Third, even in the hands of experts, several of these readings are somewhat subjective by the evaluator and tend to vary in accuracy not only from one evaluator to the next, but by the best of examiners. Equally important is the fact that these clinical parameters cannot determine current status of the disease, and a significant amount of damage must occur before these diagnostic parameters are able to detect a sufficient level of disease. Our hypothesis was that a combination of salivary analytes that relate to the biological processes of periodontitis will effectively discriminate this destructive disease from gingival inflammation and periodontal health. Therefore, the possibility of a quick, easy and reliable method of assessing and monitoring periodontal disease should provide important diagnostic information that improves and speeds treatment decisions and moves the field closer to individualized point-of-care diagnostics.

## Materials and methods

These case-control studies were conducted at the University of Kentucky College of Dentistry from 2009 through 2013. The protocols were approved by the Institutional Review Board at the University of Kentucky (12-0673-F2L; 04-0339-F1V; 10-0615-F6A; 07-0780-F6A). Participants were recruited from the general clinic and student populations of the College of Dentistry. Two hundred and nine persons participated some of whom have been described in previous reports (Thomas et al., 2009; Al-Sabbagh et al., 2012; Syndergaard et al., 2014). Each participant was given verbal and written information that described the nature of the study, and each signed informed consent prior to enrollment of the study. Inclusion criteria included subjects older than 18 years of age who were in good general health (excluding the case definition) and had a minimum of 20 teeth.

Individuals were excluded from either group if there was evidence of alcoholism, liver, kidney, or salivary gland dysfunction, inflammatory bowel disease, granulomatous diseases, diabetes, undergoing or had undergone organ transplant or cancer therapy, had a periodontal abscess or had previous treatment for periodontal disease or aggressive periodontitis. Pregnancy or lactation, use of antibiotics or immunosuppressant medication within the last 1 month, need for antibiotics for infective endocarditis prophylaxis during dental procedures, symptoms of acute illness (i.e., fever, sore throat, body aches, and diarrhea), removable prosthodontic or orthodontic appliances or presence of an oral mucosal inflammatory condition (e.g., aphthous, lichen planus, leukoplakia, and oral cancer) also were exclusion criteria.

### Clinical evaluation

All subjects received a full mouth periodontal examination. The medical and dental history was obtained and reviewed along with exclusion criteria prior to the periodontal examination. Findings from the head, neck, and oral examination were recorded as being normal or abnormal. All clinical findings were recorded on data collection worksheets. PPD was measured at six locations per tooth (mesial-buccal, mid-buccal, distal-buccal, mesial lingual, mid-lingual, and distal-lingual) using a PCP-UNC 15 probe. After the measurement of PPDs, all sites were observed for BOP (Thomas et al., 2009; Sexton et al., 2011; Al-Sabbagh et al., 2012; Syndergaard et al., 2014). CAL was also determined at all six locations per tooth. The percentage of sites affected with BOP and PPD were calculated by taking the number of sites affected divided by the total number of sites present for each subject. Healthy patients were categorized by BOP at ≤10% of sites (6 sites per tooth), < 3% of sites with PPD ≥ 4 mm, and no sites with clinical attachment loss (CAL) ≥ 2 mm. Subjects in the gingivitis group were defined as BOP at ≥20% of sites, < 3% of sites with PPD ≥ 4 mm, and no sites with CAL ≥ 2 mm. The periodontitis group had BOP at >10% of sites, with >5% of sites with PPD ≥ 4 mm and CAL ≥ 2 mm.

### Salivary samples

Saliva samples were collected from both groups prior to clinical evaluation. All subjects rinsed with tap water (10 mL) for 30 s and expectorated prior to saliva collection. Unstimulated whole saliva was collected according to a modification of the method described by Navazesh (1993). Subjects were asked to avoid oral hygiene measures (i.e., flossing, brushing, and mouth rinses), eating, drinking, or gum chewing 1 h prior to saliva collection. Subjects then expectorated at least 5 mL of unstimulated whole saliva into sterile tubes containing lyophilized protease inhibitor solution (SIGMAFast). Saliva samples were collected on ice. Aliquots were prepared and samples were frozen at -80°C until analysis.

### Salivary molecular biomarkers

The MILLIPLEX MAP Kit (EMD Millipore, Billerica, MA, USA) was used to detect IL-1β, IL-6, MMP-8, and MIP-1α. This kit was used to analyze individual saliva samples for the four analytes using a Luminex 100IS instrument (EMD Millipore) according to the manufacturer's instructions. All analyses were performed in duplicate within 6 months of obtaining the sample. Standards were included on all runs, and all results are reported within the linearity of the assays.

### Statistical analyses

Descriptive statistics were calculated for the demographic data and individual salivary anaytes. An ANOVA was used to evaluate differences in levels across the three groups with Tukey's *post-hoc* testing (SigmaStat v3.5, San Jose, CA). A Pearson Correlation analysis was conducted to relate levels of the salivary analytes to clinical features of the population. Finally, Chi-square test and Relative Risk ratio was determined using individual analytes to discriminate periodontitis from health or gingivitis (MedCalc, v14.12, Ostend, Belgium).

To determine the optimal cut points for distinguishing between the periodontitis and non-periodontitis patients (healthy and gingivitis) the analytes were used individually as predictors in a linear regression model (SAS v9.4, Cary, NC). The intersection of the sensitivity and specificity was used as the optimal cut point for the predictors. The AUC was also calculated for each individual analyte.

Four different classification techniques namely [Linear Discriminant Analysis (LDA), Quadratic Discriminant Analysis (QDA), Naïve Bayes Classifier (NB), Support Vector Machine (SVM)] were used to discern gingivitis from periodontitis and health from periodontitis using the salivary markers. Classification performance measures (sensitivity, specificity and accuracy) were estimated using the clinical labels of the samples as the ground truth and leave-ten-out cross-validation where 10 samples are set aside as the test set with the remaining samples as the training set. The mean values of the classification performance measures were estimated across (*N* = 100) independent realizations by randomly assigning the samples to the test and training sets. Classification was repeated using all possible combinations of markers (pairs, triplets, and all four markers).

## Results

### Distribution of analytes in saliva

Included in the cohort were 65 subjects in the healthy group, 43 subjects in the gingivitis group and 101 subjects in the periodontitis group, with some differences in age, gender, race/ethnicity, and smoking across the groups (Table 1).

**Table 1:** **Demographics of the population**.

	**Healthy (*n* = 65)**	**Gingivitis (*n* = 43)**	**Periodontitis (*n* = 101)**
Age (years; mean ± SD)	28.2 ± 5.9^a^	27.8 ± 4.5^a^	42.0 ± 10.4^a^
Female (%)	60.0^a^	48.8^a^	32.7^a^
White (%)	90.0^a^	95.4^a^	41.60^a^
Hispanic (%)	2.5	2.3	34.7
African American (%)	0	2.3	17.8
Asian/Other (%)	7.5	0	6.9
Current tobacco use (%)	0^a^	0^a^	28.0^a^
No. of teeth	27.6 (range 20–32)	27.6 (range 24–28)	27.1 (range 20–28)
**PERIODONTAL INDICES (%SITES; MEAN± SD)**			
BOP sites	4.26 ± 3.4^a^^,^^b^	25.4 ± 5.9^a^^,^^b^	57.4 ± 24.2^a^
PD ≥ 4 mm sites	0.6 ± 1.1^a^	0.6 ± 2.0^a^	27.3 ± 14.7^a^
PD ≥ 5 mm sites	0.1 ± 0.3^a^	0.1 ± 0.2^a^	16.6 ± 11.2^a^

a *Significantly different from periodontitis group at least at p < 0.05*.

b *Significantly different from healthy group at least at p < 0.05*.

**Figure 1:**
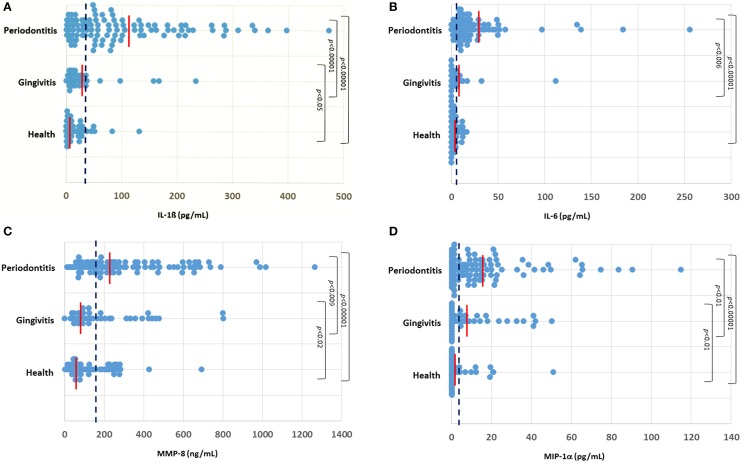
**Distribution of levels of IL-1ß (A), IL-6 (B), MMP-8 (C), and MIP-1α (D) in the three groups of subjects**. Each point denotes the analyte value for a patient. The vertical red line denotes the group mean and the vertical blue dashed line denotes the threshold cutoff for each analyte (IL-1ß ≥ 28 pg/mL; IL-6 ≥ 5.5 pg/mL; MMP-8 ≥ 140 ng/mL; MIP-1α ≥ 5 pg/mL).

The profiles of salivary analytes IL-1ß, IL-6, MMP-8, and MIP-1α in the cohort are displayed in Figure 1. IL-1β concentrations were significantly higher in the periodontitis group (102.3 ± 10.1 SEM pg/mL) compared to levels in the gingivitis (28.7 ± 7.3) and healthy (14.6 ± 2.6) subjects. A minimal difference was noted in the cytokine comparing the overall gingivitis group to the healthy individuals. Similarly, a significantly elevated concentration of salivary IL-6 was found in the periodontitis group (22.8 ± 3.7 pg/mL) compared to both the gingivitis (6.3 ± 2.7) and healthy (3.7 ± 0.5) subjects. Consistent with this, IL-6 levels were below the level of detection in the assay (0.64 pg/mL) in approximately 2% of the periodontitis patients, compared with nearly 20% of both gingivitis and healthy subjects. MMP-8 levels were also significantly elevated in the periodontitis group (314.1 ± 25.5 ng/mL) compared to both gingivitis (199.0 ± 29.1) and healthy (130.7 ± 14.5) subjects, although there appeared to be a greater overlap across the groups with this analytes vs. the others that were examined. Finally, MIP-1α was significantly increased in the periodontitis group (16.2 ± 2.2 pg/mL) compared to both gingivitis (12.0 ± 2.2) and health (3.2 ± 1.0) groups.

### Salivary analytes and disease measures

**Figure 2:**
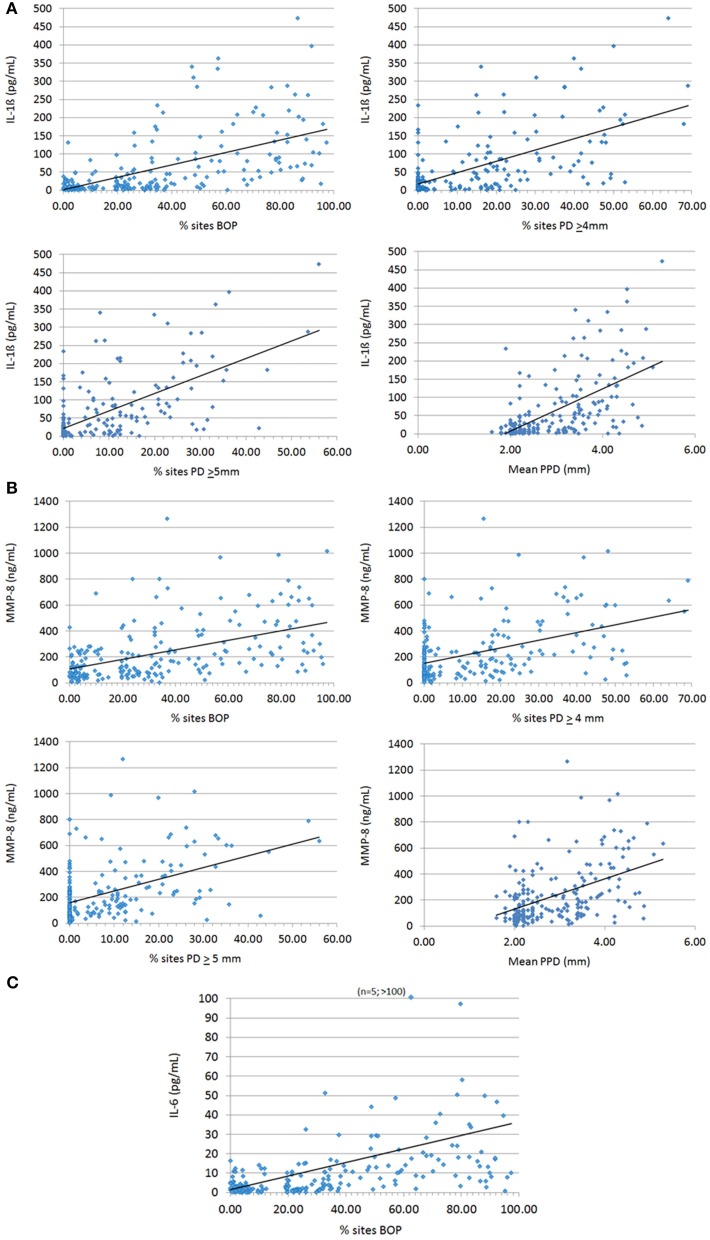
**Correlation analyses of biomarkers IL-1ß (A), MMP-8 (B), and IL-6 (C) significantly related to clinical indices of periodontitis**. Each point denotes a patient in the population (*n* = 209) and the line denotes the correlation trendline.

The relationship of these salivary biomarkers with the clinical parameters of oral disease was examined in an attempt to identify any clinical and biological relationships. Across the spectrum of patents, IL-1ß, IL-6, and MMP-8 levels were significantly positively correlated with BOP frequency in the population, while both IL-1ß and MMP-8 were significantly correlated with percent sites with advanced PPDs across the population (Figure 2). Table 2 shows the results describing that only IL-1ß and MMP-8 levels were significantly correlated with PPD levels across the population and that this relationship was also observed in the periodontitis patients to a similar degree with the extent of disease (i.e., %sites with ≥4 mm or ≥5 mm PPD) and the severity of disease (i.e., mouth mean PPD).

**Table 2:** **Correlation of individual salivary biomarkers with bleeding on probing and frequency of probing pocket depths in the population**.

**Clinical measure**	**Analyte**	**Total**	**Health**	**Gingivitis**	**Periodontitis**
%BOP > 0	**IL-1ß**	**0.6144**	−0.0311	−0.0719	**0.4669**
%PPD ≥ 4 mm		**0.6429**	−0.1219	−0.0006	**0.5167**
%PPD ≥ 5 mm		**0.5836**	0.0356	0.2142	**0.4068**
Mean PPD		**0.6009**	0.0757	−0.1269	**0.4596**
%BOP > 0	**IL−6**	**0.3622**	0.0156	−0.0936	0.2098
%PPD ≥ 4 mm		0.2887	0.0848	−0.0136	0.0611
%PPD ≥ 5 mm		0.3163	−0.0146	0.0835	0.1305
Mean PPD		0.3247	0.1312	0.3292	0.0861
%BOP > 0	**MMP−8**	**0.4841**	0.1657	0.2583	**0.3827**
%PPD ≥ 4 mm		**0.4502**	−0.1248	−0.00794	**0.3613**
%PPD ≥ 5 mm		**0.4590**	−0.1182	−0.1395	**0.3746**
Mean PPD		**0.4629**	0.0733	−0.0602	**0.3862**
%BOP > 0	**MIP−1α**	0.3280	−0.0308	−0.1141	0.1641
%PPD ≥ 4 mm		0.2821	−0.1540	−0.0136	0.1179
%PPD ≥ 5 mm		0.2883	−0.0958	−0.0691	0.1449
Mean PPD		0.2942	−0.3365	0.1428	0.1130

*Figures in bold denote significant correlation at least at p < 0.05. Total denotes all 3 subgroups comprising the entire cohort*.

### Discriminatory analytes and periodontal disease

Data were then used to explore the capacity of these four analytes to effectively discriminate periodontitis from gingivitis and health. Three strategies were used in this approach. First threshold cutoff values for each analyte were based upon the population distribution for IL-1ß (≥28 pg/mL), IL-6 (≥5.5 pg/mL), MMP-8 (≥140 ng/mL), and MIP-1α (≥5 pg/mL) and selected to optimize sensitivity for detection of periodontitis. Based upon these threshold values, the results in Table 3 show that for elevated concentrations of IL-1ß, IL-6, and MMP-8 significantly categorized periodontitis patients compared with both healthy and gingivitis groups. These data also provided an assessment of the relative risk for a patient to be clinically classified as periodontitis based upon the individual biomarkers, with each marker showing a very high level of significance and a 2 to 4-fold relative risk when the concentration was above the threshold.

**Table 3:** **Discriminatory power of individual analytes using thresholds based on responses across the population**.

**Analyte**	**Healthy (*n* = 65)**	**Gingivitis (*n* = 43)**	**Periodontitis (*n* = 101)**	**Periodontitis vs. health**	**Periodontitis vs. gingivitis**	**Relative risk**
IL-1ß	9^a^	10	76	*X*^2^ = 57.24 *P* < 0.0001	*X*^2^ = 31.76 *P* < 0.0001	4.2772 (2.8013–6.5309) *P* < 0.0001
IL-6	10	10	78	*X*^2^ = 58.26 *P* < 0.0001	*X*^2^ = 34.73 *P* < 0.0001	4.1703 (2.7689–6.2810) *P* < 0.0001
MMP-8	22	18	72	*X*^2^ = 21.07 *P* < 0.0001	*X*^2^ = 9.92 *P* = 0.0016	1.9440 (1.4722–2.5583) *P* < 0.001
MIP-1α	7	12	42	*X*^2^ = 34.6 *P* < 0.0001	*X*^2^ = 2.15 NS	2.3637 (1.4787–3.7785) *P* = 0.0003

a *Denotes number of patient samples above the thresholds of: IL-1ß—28 pg/mL, IL-6—5.5 pg/mL, MMP-8—140 ng/mL, MIP-1α—5 pg/mL*.

**Figure 3:**
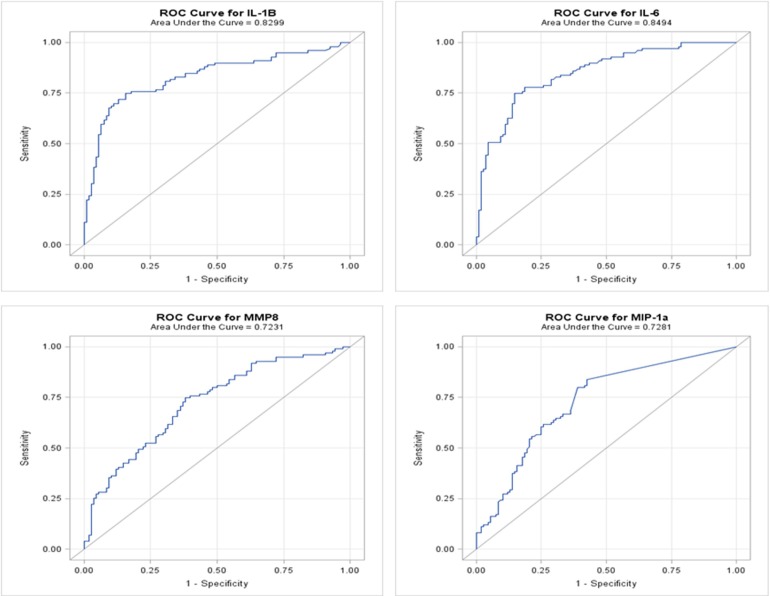
**ROC analysis of individual salivary analytes in the entire population, comparing levels in periodontitis to those in “not periodontitis” (i.e., health and gingivitis)**.

We developed ROC curves and determined the AUC (c-statistic) for each analyte individually (Figure 3) by comparing the levels in the periodontitis group vs. those identified in both health and gingivitis. The summary results in Table 4 show the highest sensitivity and specificity for periodontitis with both IL-1ß and IL-6, paralleling the results found using our earlier approaches to stratify the analyte levels with disease.

**Table 4:** **Results from ROC logistic regression analysis of individual salivary biomarker levels comparing periodontitis group to “not periodontitis” (i.e., health and gingivitis) group**.

**Analyte**	Optimal cut^*^	**Sensitivity**	**Specificity**	**PPV**	**NPV**	**AUC**
IL-1β	24.00 pg/mL	0.752	0.759	0.745	0.766	0.830
IL-6	5.11 pg/mL	0.780	0.787	0.772	0.794	0.849
MMP8	165.92 ng/mL	0.653	0.667	0.647	0.673	0.728
MIP-1α	3.28 pg/mL	0.663	0.676	0.657	0.682	0.723

* *The optimal cut was selected based on the intersection of the specificity and sensitivity plots*.

Finally, in recognizing the capacity of ROC analyses to potentially overfit the classification properties of the data (Baker, 2003), we utilized four classification techniques (LDA, QDA, NB, SVM) to evaluate the capacity of various groupings of analytes to effectively categorize the periodontitis patients. Table 5 provides the classification performance measures (sensitivity, specificity, accuracy) using the LDA approach. Outcomes from QDA, NB, and SVM were similar hence not shown. The LDA results were consistent with the ROC analyses and clearly showed that combinations of these biomarkers improved the sensitivity, specificity, and accuracy of the identification of periodontitis vs. either health or gingivitis subjects when compared to the performance of the individual biomarkers. As might be expected, the greatest sensitivity, specificity, and accuracy were identified in comparing health with periodontitis patients. The classification performance measures using the pair (IL-1ß, IL-6) was especially pronounced indicating that these molecular markers play a critical role in discerning periodontitis from gingivitis and health.

**Table 5:** **Average Classification performance measures (Sensitivity, Specificity, Accuracy) estimated across all possible combinations of markers using leave-10-out cross-validation and 100 independent realizations**.

	**IL-1ß IL-6**	**IL-1ß MMP-8**	**IL-1ß MIP-1α**	**IL-6 MMP-8**	**IL-6 MIP-1 α**	**MMP-8 MIP-1 α**	**IL-1ß IL-6 MMP-8**	**IL-1ß IL-6 MIP-1 α**	**IL-6 MMP-8 MIP-1 α**	**IL-1ß IL-6 MMP-8 MIP-1 α**
**HEALTH vs. PERIODONTITIS**										
Sensitivity	0.8115	0.7798	0.7965	0.8103	0.7878	0.7010	0.7780	0.8055	0.7948	0.7868
Specificity	0.7718	0.7675	0.7520	0.7283	0.7720	0.7728	0.7708	0.7868	0.7430	0.7820
Accuracy	0.7916	0.7736	0.7743	0.7693	0.7799	0.7369	0.7744	0.7961	0.7689	0.7844
**GINGIVITIS vs. PERIODONTITIS**										
Sensitivity	0.7755	0.7630	0.7545	0.7843	0.8073	0.6473	0.7653	0.7830	0.7260	0.7803
Specificity	0.7565	0.7323	0.7865	0.7115	0.7073	0.6190	0.7538	0.7905	0.7340	0.7810
Accuracy	0.7660	0.7476	0.7705	0.7479	0.7573	0.6331	0.7595	0.7868	0.7300	0.7806

## Discussion

Studies over the last two decades have provided an array of targets for detection of various substances in saliva (Desai and Mathews, 2014), including drugs of abuse (Moore and Crouch, 2013), alcohol (Swift, 2003), estradiol (Lewis, 2006), cotinine (Scheidweiler et al., 2011), cortisol (van Andel et al., 2014), and HIV antibody (Pant Pai et al., 2012) as examples. Based upon the potential value of saliva as a non-invasive screening tool for oral disease(s), this study focused on the quantification of a group of analytes that may act as biomarkers for periodontitis and aid in the development of personalized approaches for periodontal risk assessment. Movement toward an era of personalized medicine and individualized clinical decisions in periodontology requires significant improvement in our ability to define risk and predict disease progression. While the medical field routinely makes clinical diagnoses based on signs and symptoms (e.g., pneumonia, diarrhea), decisions on patient management and treatment do not stop here. Modern medicine integrates these clinical descriptors with biological assessments that enable the physician to focus on the specific disease etiology and unique features of the patient in finalizing a treatment strategy. However, it is clear the clinical measures alone do not provide sufficient information to determine which patients will/won't progress, and what therapy should be provided to those at risk. Recent investigations using proteomic and transcriptomic analyses have dramatically expanded the potential panel of biomarkers for gingivitis; however, generally these studies have been limited to comparisons of gingivitis with health, or periodontitis with health (Offenbacher et al., 2009; Jönsson et al., 2011). No studies have been published that extrapolate from these reports analytes in gingival tissues, crevicular fluid, or saliva that discriminate gingivitis from periodontitis.

Periodontitis represents a persistent inflammatory response to chronic biofilms inhabiting the subgingival crevice (Hajishengallis, 2014; Nibali et al., 2014). The current paradigm suggests that variations in the quantity and quality of the oral microbial ecology at health, gingivitis, and periodontitis sites results in a dysregulated inflammatory response that causes release of a variety of host biomolecules that lead to the clinical features of periodontitis. These biomolecules can represent the various stages of progression of the destructive inflammatory response, including IL-1ß as a proinflammatory cytokine that has effects on coupling processes in bone biology (Nakashima and Takayanagi, 2009; Braun and Schett, 2012), IL-6 as a pleiotropic cytokine that communicates inflammatory signals with a number of cell types, and can elicit bone resorptive processes (Huang et al., 2001; Braun and Schett, 2012), MMP-8 a primary collagenase effective on both types I and III collagen and released by neutrophils that alters the integrity of soft tissues in the periodontium (Salminen et al., 2014), and MIP-1α (also known as CCL3), a chemokine macrophage inflammatory protein that binds to CCR1, CCR4, and CCR5 receptors frequently on the surface of immune cells, recruiting them into sites of inflammation (Kabashima et al., 2001). This chemokine has also been found to activate osteoclasts, particularly related to bone resorption in multiple myeloma through these receptors (Terpos et al., 2005). Importantly, many of these biomarkers have been detected in saliva and correlate with periodontal disease. While it is clear that these biomolecules contribute to the inflammatory and tissue destructive processes of periodontitis, a number of them have also been detected in serum associated with chronic inflammation related to systemic diseases (Fain, 2006; Zakynthinos and Pappa, 2009; Wu et al., 2010; Cierny et al., 2014). We have also evaluated some of these in saliva and while they can be elevated to some degree with systemic inflammation vs. control individuals, the levels of these analytes in saliva with periodontitis are significantly increased compared to any of the systemic conditions (Mirrielees et al., 2010; Miller et al., 2014). Nevertheless, a clinical medical history should be taken into account to minimize false-positive responses in periodontally healthy subjects.

IL-1ß has been identified in gingival crevicular fluid (GCF; Faizuddin et al., 2003; Kinney et al., 2014) and saliva (Miller et al., 2006; Yoon et al., 2012; Salminen et al., 2014) in elevated levels in numerous investigations of periodontitis. Some of these studies have also shown that the levels are related to disease extent/severity (Tobón-Arroyave et al., 2008) and decrease with therapy (Sexton et al., 2011). Moreover, while it is somewhat controversial at this time, data exist suggesting that polymorphisms in this gene impose a risk for periodontitis (Lang et al., 2000; Lee et al., 2012; Diehl et al., 2015). We have previously identified elevated levels of IL-1ß in saliva from periodontitis patients compared to orally healthy individuals (Miller et al., 2006, 2014; Frodge et al., 2008), and have shown a relatively stable level of this analyte in whole saliva of healthy subjects over time (Thomas et al., 2009; Syndergaard et al., 2014). The findings in this study extended these results by inclusion of gingivitis patients and demonstrating significant elevation in periodontitis saliva and positive correlations of salivary IL-1ß levels with BOP and measures of pocket PPD. Determining a threshold for positive response at ≥28 pg/mL showed a significant discriminatory power in periodontitis for this analyte, with a RR = 4.2772 for periodontitis with elevated salivary IL-1ß. This finding is consistent with previous results reported by us and others; however, these reports generally compared periodontitis to oral health (Miller et al., 2006; Gursoy et al., 2009; Kaushik et al., 2011; Kinney et al., 2014; Salminen et al., 2014).

Increased IL-6 levels have also been found in GCF (Fujita et al., 2012; Javed et al., 2012) and saliva (Costa et al., 2010; Gümüs et al., 2014; Javed et al., 2014) from periodontitis patients vs. health controls. A limited number of studies have also identified increases in IL-6 in periodontitis tissues (Duarte et al., 2012), although a meta-analysis by Song et al. (2013) suggested genetic polymorphisms for this cytokine may be limited in their relationship to periodontitis across various populations. Treatment studies have also documented decreases in IL-6 in GCF following non-surgical periodontal therapy (Kardesler et al., 2011; de Lima Oliveira et al., 2012). The overall levels of this cytokine were substantially lower than IL-1ß, and nearly 25% of the healthy/gingivitis groups showed no detectable IL-6 in saliva and only 2/101 periodontitis patients. As such, the levels were significantly elevated in the periodontitis group. Minimal correlations in IL-6 levels across the population were observed, appearing only related to BOP levels. However, using a threshold value of = 5.5 pg/mL, showed a significant increase in positive responses in the periodontitis patients with a highly significant RR = 4.1703 for elevated salivary IL-6 levels commensurate with periodontitis.

MMP-8, a major factor produced by neutrophils at sites of inflammation has a robust literature demonstrating elevations in GCF (Tervahartiala et al., 2000; Mäntylä et al., 2006; Kinney et al., 2014) and saliva (Javed et al., 2014; Salminen et al., 2014) in periodontitis. Sorsa et al. (1999) have demonstrated the value of detection of elevations in this analyte for diagnosing periodontitis and following therapeutic intervention for the disease (Kinane et al., 2003; Sexton et al., 2011). We had confirmed these types of findings in periodontitis and healthy groups (Miller et al., 2006), but demonstrate in this study differences also between periodontitis and gingivitis patients. Interesting aspects of the distribution of MMP concentrations was the large range in values detected in saliva from each of the groups, and that the gingivitis group showed significantly higher levels than healthy subjects. This was not totally unexpected due to the inflammation in the gingivitis patients. This finding was also supported by the significant positive correlation of MMP-8 levels with all of the clinical measures. Identifying a threshold of ≥140 ng/mL demonstrated a significantly greater frequency of positive responses in periodontitis with an RR = 1.9440.

Of the analytes targeted in this study, MIP-1α has a least amount of information regarding its distribution in periodontitis. MIP-1α (CCL3) is a member of the cysteine-cysteine chemokine family which is secreted by macrophages, neutrophils, basophils, dendritic cells, lymphocytes and epithelial cells and mediates granulocyte migration and adhesion (Kabashima et al., 2001, 2002; Glass et al., 2003). It is an upstream signaling protein that stimulates monocytes and/or osteoclast progenitor cells to become active osteoclasts in a RANK/RANKL and dose-dependent manner (Giuliani et al., 2004). MIP-1α has been detected at higher salivary levels (50-fold) in a longitudinal study of seven adolescents who had aggressive periodontitis compared with controls (Fine et al., 2009), and appeared to increase prediction of disease progression. We had shown previously that MIP-1α was significantly higher in periodontitis subjects compared to healthy individuals and decreased following periodontal therapy (Sexton et al., 2011; Al-Sabbagh et al., 2012). These findings suggested that the salivary level of MIP-1α could have clinical utility as a screening tool for moderate to severe periodontal disease. However, as we noted its utility for discriminating between intermediate levels of disease (gingivitis, mild periodontitis) and health was indeterminant. This study expanded the target population and identified additional critical features of this salivary analyte. First, concentrations in periodontitis were significantly increased compared to both health and gingivitis groups. Second, correlation with the various clinical parameters was observed with the entire population. Finally using a threshold of ≥5 pg/mL demonstrated a significantly increased frequency of being classified as periodontitis with an RR = 2.3637.

While each of these analytes appeared useful in discriminating periodontitis from health and gingivitis, the combination of sensitivity, specificity, and accuracy was improved by exploring combinations of the biomarkers. Our data showed that concentrations above a defined threshold for any three of the biomarkers identified 67.3% of periodontitis, 18.6% of gingivitis, and 6.2% of healthy subjects. We then identified that pairs of markers, including IL-1ß/IL-6, IL-1ß/MMP-8, and IL-6/MMP-8, provided an increase in diagnostic ability by demonstrating sensitivity and specificity values approximating 0.8. We also identified that inclusion of three biomarkers into the biologic diagnostic model, IL-1ß/IL-6/MMP-8, yielded a small increase in the sensitivity, specificity, and accuracy values. Using MIP-1α in these various groupings provided little improvement in the discriminatory characteristics to identify periodontitis patients. Finally, our diverse analyses allowed us to provide potential thresholds to discriminate periodontitis (i.e., IL-1ß: 24–28 pg/mL; IL-6: 5.11–5.5 pg/mL; MIP-1α: 3.28–5 pg/mL, and MMP-8: 140–165.9 ng/mL).

While these analytes have been evaluated as single biomarkers in saliva for periodontitis in other studies, generally these previous studies have not evaluated combinations of biomarkers representing the various disease processes that occur in periodontitis, nor have they included patients with gingivitis to elucidate the effect of gingival inflammation on these analytes. This cross-sectional study enabled a “head-to-head” comparison of these salivary analytes in destructive (periodontitis) and non-destructive reversible (gingivitis) gingival inflammation. Thus, we can conclude from these studies that select biomarkers, particularly in combination provide enhanced sensitivity and specificity for identification of periodontitis in the population. Also, of important note, as can be seen in the salivary analyte distribution graphs, there was a subset of gingivitis patients and even a few healthy subjects that demonstrated elevated levels of one or more of these salivary analytes. Generally, when these elevated responses occurred, there were multiple analyte elevations in the same individuals. Historically, evaluation of salivary biomarker data was structured to “force” the patients into a specific clinical group and accept that this “within group heterogeneity” would be reflected in the variation in analyte levels contributing to group differences. However, in this era of personalized and precision medicine (Hood et al., 2012; Mirnezami et al., 2012; Flores et al., 2013; Schmidt, 2014), we submit that there is substantive value in identifying these subsets of individuals within the larger clinical groupings and document unique features of their disease trajectory and/or patient specific responses that could characterize risk or resistance to disease, and/or response to therapy.

### Conflict of interest statement

The authors declare that the research was conducted in the absence of any commercial or financial relationships that could be construed as a potential conflict of interest.
